# Association of citrulline concentration at birth with lower respiratory tract infection in infancy: Findings from a multi-site birth cohort study

**DOI:** 10.3389/fped.2022.979777

**Published:** 2022-10-17

**Authors:** Brittney M. Snyder, Tebeb Gebretsadik, Kedir N. Turi, Christopher McKennan, Suzanne Havstad, Daniel J. Jackson, Carole Ober, Susan Lynch, Kathryn McCauley, Christine M. Seroogy, Edward M. Zoratti, Gurjit K. Khurana Hershey, Sergejs Berdnikovs, Gary Cunningham, Marshall L. Summar, James E. Gern, Tina V. Hartert, on behalf of the ECHO-CREW investigators

**Affiliations:** ^1^ Department of Medicine, Vanderbilt University Medical Center, Nashville, TN, United States; ^2^ Department of Biostatistics, Vanderbilt University Medical Center, Nashville, TN, United States; ^3^ Department of Statistics, University of Pittsburgh, Pittsburgh, PA, United States; ^4^ Department of Public Health Sciences, Henry Ford Health System, Detroit, MI, United States; ^5^ Department of Pediatrics, University of Wisconsin, Madison, WI, United States; ^6^ Department of Human Genetics, University of Chicago, Chicago, IL, United States; ^7^ Department of Medicine, University of California, San Francisco, CA, United States; ^8^ Department of Internal Medicine, Henry Ford Health System, Detroit, MI, United States; ^9^ Division of Asthma Research, Cincinnati Children's Hospital Medical Center, Cincinnati, OH, United States; ^10^ Department of Pediatrics, University of Cincinnati College of Medicine, Cincinnati, OH, United States; ^11^ Department of Medicine, Northwestern University, Chicago, IL, United States; ^12^ Department of Genetics and Metabolism, Children’s National Medical Center, Washington, DC, United States; ^13^ Department of Pediatrics, Vanderbilt University Medical Center, Nashville, TN, United States

**Keywords:** lower respiratory tract infection, infancy, newborn screening (NBS), metabolites, citrulline

## Abstract

Assessing the association of the newborn metabolic state with severity of subsequent respiratory tract infection may provide important insights on infection pathogenesis. In this multi-site birth cohort study, we identified newborn metabolites associated with lower respiratory tract infection (LRTI) in the first year of life in a discovery cohort and assessed for replication in two independent cohorts. Increased citrulline concentration was associated with decreased odds of LRTI (discovery cohort: aOR 0.83 [95% CI 0.70–0.99], *p* = 0.04; replication cohorts: aOR 0.58 [95% CI 0.28–1.22], *p* = 0.15). While our findings require further replication and investigation of mechanisms of action, they identify a novel target for LRTI prevention and treatment.

## Introduction

Lower respiratory tract infections (LRTIs) are the leading cause of mortality in young children worldwide (1). While children with underlying conditions, such as prematurity, congenital heart disease, and chronic lung disease, are at highest risk of severe LRTI, the majority of these events, including hospitalizations, occur in previously healthy children (2, 3). In the first year of life, LRTIs are primarily caused by respiratory viral pathogens, most commonly respiratory syncytial virus (RSV) (4). With the exception of antiviral agents for influenza and SARS-CoV-2, most infections are not currently vaccine preventable and treatment is only supportive care (5). A very small subset of premature infants with significant pulmonary and cardiac conditions may qualify for RSV immunoprophylaxis (6). Despite the significant public health burden of LRTIs, effective treatment and prevention strategies are lacking for most infants (5).

Energy metabolism is a highly regulated process which plays an important role in susceptibility to viral infections (7). As viruses rely upon energy produced by host cell metabolism to replicate, metabolic pathways influence virulence (7, 8). Immune cells also depend upon nutrients produced through cellular metabolism to recognize and respond to pathogens (9). Immune responses are impaired in individuals with metabolic disorders, allowing viruses to further compromise the immune system and enhance metabolic dysfunction (10, 11). Therefore, assessing the association between the variability in normal metabolism and viral infection severity may provide important insights on LRTI pathogenesis, may aid in the identification of those at risk, and identify new pathways that may modify immune regulation that could be targeted for prevention and treatment. Our objective was to test the association of metabolites measured at birth with risk of LRTI during the first year of life using targeted metabolic data from newborn screening (NBS) programs.

## Materials and methods

### Study design, populations, and data collection

This multi-site cohort study included three birth cohorts from the NIH Environmental influences on Child Health Outcomes (ECHO) Children's Respiratory and Environmental Workgroup (CREW) consortium (12). We utilized a population-based birth cohort which was the largest of the three cohorts (INSPIRE, *n* = 1949) for the discovery phase and the smaller cohorts (MAAP, *n* = 141; WISC, *n* = 270) to replicate findings. We included enrolled infants with at least one year of follow-up whom we linked with NBS blood metabolic data, including targeted measurement of amino acids, free carnitine, and acylcarnitines. Each state's public health department is responsible for deciding which conditions, and corresponding metabolites, are included on the NBS panel based on evidence of net benefit of screening, availability of effective treatments, and screening capability of the state. Most states screen for the panel of conditions recommended by the U.S. Health Resources / Services Administration, using the same methodology (13), while some states additionally screen for newer conditions (14, 15).

Our primary exposures were metabolite concentrations at birth. Metabolic data for newborns were obtained from the NBS programs at the Tennessee Department of Health, Michigan Department of Health and Human Services, and Wisconsin State Laboratory of Hygiene. Data were provided for infants who did not screen positive for any inherited disorder [i.e., metabolite concentrations were within the normal range, representing >99% of infants in the US (16)] to reduce the risk of potential participant identification and remove skewed metabolite profiles due to inborn errors of metabolism. These data were then linked with demographic and clinical data from each of the cohorts. Metabolites measured in each cohort are listed in Supplementary Table S1. Our primary outcome, LRTI, was ascertained by parental report, physician diagnosis, or medical record documentation of bronchiolitis or pneumonia at any time during the first year of life and defined at age one-year dichotomously as LRTI yes or no. Data collection for ascertainment of the primary outcome within each cohort is summarized in Supplementary Table S2. Demographic and clinical characteristics were ascertained from questionnaires administered during the first year of life.

### Patient consent statement

The protocol and informed consent documents were approved by the Vanderbilt University Medical Center, Henry Ford Health System, University of Wisconsin, Tennessee Department of Health, Michigan Department of Health and Human Services, and Wisconsin Department of Health Services Institutional Review Boards. Written informed consent or parent's/guardian's permission was obtained, along with child assent as appropriate, for CREW participation and for participation in specific cohorts.

### Statistical analysis

We compared demographic and clinical characteristics between the cohorts using Kruskal-Wallis or Pearson *χ*^2^ test, as appropriate. Our *a priori* statistical plan utilized a common, pre-specified two-stage procedure (17) to identify LRTI-related metabolites in the discovery cohort (Supplementary Figure S1). In the first stage, we used elastic net to identify leading metabolites associated with LRTI in the first year of life. Elastic net is a penalized regression method for selecting groups of correlated metabolites while performing variable selection and continuous shrinkage (18). We included all metabolites in one logistic regression model, which helped reduce the multiple metabolite testing burden. Although there was some skewness and zero values, metabolite concentrations were generally well distributed (normally) (Supplementary Figure S2). As having normal distributions is not an assumption of logistic regression, we utilized raw, untransformed metabolite concentrations in the analyses.

In the second stage, we evaluated the selected metabolites in a multivariable logistic regression model simultaneously, while adjusting for *a priori* selected demographic and clinical characteristics previously shown to be associated with metabolism or infant LRTI. We calculated each metabolite's adjusted odds ratio for an interquartile range [IQR] difference increase in its concentration. For each metabolite that remained significantly associated with LRTI after the second stage (*p* < 0.05), we tested its association with LRTI in the replication cohorts using meta-analyzed logistic regression. In the replication analysis, our regression power was limited by sparse LRTI events. To avoid overfitting, we decided *a priori* to limit covariate adjustment to infant sex (estimated power in replication cohorts: 6%–25%; see Supplementary Material for details on power calculation).

To provide additional insight into pathways involved in severity of RSV infection, we performed a sub-analysis assessing the association between each metabolite identified in the primary analysis and severity of RSV infection. We performed this analysis among a subset of infants in the discovery cohort with either RSV upper respiratory tract infection (URTI, less severe) or RSV LRTI (more severe) identified through biweekly surveillance during RSV season and serology at one year. We used multivariable logistic regression, adjusting for the same covariates included in stage two of the primary analysis, to assess the association. Data analyses were performed using R software, versions 3.6.1 and 4.0.4 (R Foundation for Statistical Computing, Vienna, Austria). Additional information on methodology can be found in the Supplementary Material.

## Results

**Figure 1:**
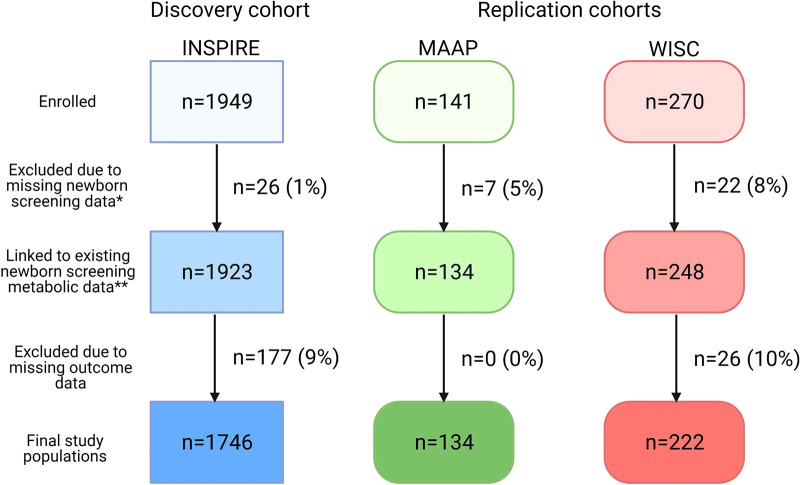
Flow diagram of study populations. Discovery cohort: INSPIRE, Infant Susceptibility to Pulmonary Infections and Asthma following RSV Exposure; Replication cohorts: MAAP, Microbes, Allergy, Asthma and Pets study and WISC, Wisconsin Infant Study Cohort. *Newborn screening data may have been missing due to refusal of newborn screening, metabolite concentrations outside the normal range, or incomplete linkage. **The study population included in the RSV sub-analysis (*n* = 912 INSPIRE infants) stemmed from this box. This figure was created with BioRender.com.

Our final study populations included 1,746 (INSPIRE, discovery cohort), 134 (MAAP, replication cohort), and 222 (WISC, replication cohort) infants after linking NBS metabolic data to >90% of infants (Figure 1). Demographic and clinical characteristics and NBS metabolite concentrations for the study populations are shown in Table 1 and Supplementary Table S3.

**Table 1:** Demographic and clinical characteristics of the study populations with linked newborn screening data and non-missing outcome data.

	INSPIRE N (%)	MAAP N (%)	WISC N (%)
**Sample size**	**1746**	**134**	**222**
Infant sex			
Male	920 (53)	64 (48)	109 (49)
Female	826 (47)	70 (52)	113 (51)
Missing	0 (0)	0 (0)	0 (0)
Infant race/ethnicity			
Non-Hispanic White	1,120 (64)	76 (57)	212 (95)^a^
Non-Hispanic Black	313 (18)	13 (10)	2 (1)
Hispanic	148 (8)	7 (5)	2 (1)
Other	165 (9)	38 (28)	6 (3)
Missing	0 (0)	0 (0)	0 (0)
Gestational age (weeks)^b^	39 (1)	39 (1)	39 (1)^a^
Missing	0 (0)	0 (0)	0 (0)
Birthweight (grams)^b^	3,442 (461)	3,484 (500)	3,408 (518)
Missing	0 (0)	0 (0)	0 (0)
Mode of delivery			
Vaginal	1,202 (69)	92 (69)	177 (80)^a^
Cesarean	544 (31)	42 (31)	45 (20)
Missing	0 (0)	0 (0)	0 (0)
Maternal smoking during pregnancy	294 (17)	8 (6)	4 (2)^a^
Missing	0 (0)	1 (1)	5 (2)
Secondhand smoke exposure during the first year of life	586 (34)	2 (1)^a^	–
Missing	0 (0)	4 (3)	222 (100)
Daycare attendance during the first year of life	580 (33)	97 (72)	160 (72)^a^
Missing	20 (1)	14 (10)	0 (0)
Maternal asthma	344 (20)	42 (31)	42 (19)^a^
Missing	1 (0)	0 (0)	3 (1)
Paternal asthma	275 (16)	23 (17)	22 (10)^a^
Missing	102 (6)	9 (7)	2 (1)
Maternal allergic rhinitis	413 (24)	36 (27)	67 (30)
Missing	1 (0)	1 (1)	2 (1)
Ever breastfed	1,402 (80)	111 (83)	209 (94)^a^
Missing	11 (1)	1 (1)	4 (2)
Maternal education			
Less than high school	130 (7)	4 (3)	0 (0)^a^
High school diploma/GED	464 (27)	16 (12)	12 (5)
Some college	523 (30)	39 (29)	59 (27)
College degree or more	629 (36)	74 (55)	146 (66)
Missing	0 (0)	1 (1)	5 (2)
Maternal marital status			
Single	700 (40)	17 (13)	8 (4)^a^
Married	1,010 (58)	116 (87)	205 (92)
Divorced/separated	36 (2)	1 (1)	3 (1)
Missing	0 (0)	0 (0)	6 (3)
Residence during the first six months of life			
Urban	1,335 (76)	134 (100)	0 (0)^a^
Rural	396 (23)	0 (0)	222 (100)
Missing	15 (1)	0 (0)	0 (0)
Living siblings		–	
0	603 (35)		65 (29)
1	545 (31)		77 (35)
≥ 2	598 (34)		79 (36)
Missing	0 (0)	134 (100)	1 (0)
Year of birth			
2012	760 (44)	N/A	N/A^a^
2013	986 (56)	N/A	4 (2)
2014	N/A	31 (23)	22 (10)
2015	N/A	74 (55)	69 (31)
2016	N/A	29 (22)	53 (24)
2017	N/A	N/A	19 (9)
2018	N/A	N/A	39 (18)
2019	N/A	N/A	16 (7)
Missing	0 (0)	0 (0)	0 (0)
Age at infant enrollment (months)^b^	2 (2)	0 (0)^c^	0 (0)^a,c^
Missing	0 (0)	0 (0)	0 (0)

GED, general educational development; N/A, not applicable (i.e., year was outside of study enrollment window).

– Data not collected.

^a^ P < 0.05 for the comparison between cohorts (in which data were collected) using Kruskal-Wallis or Pearson *χ*^2^ test, as appropriate.

^b^ Data are expressed as mean (standard deviation).

^c^ Infants were enrolled at birth.

**Figure 2:**
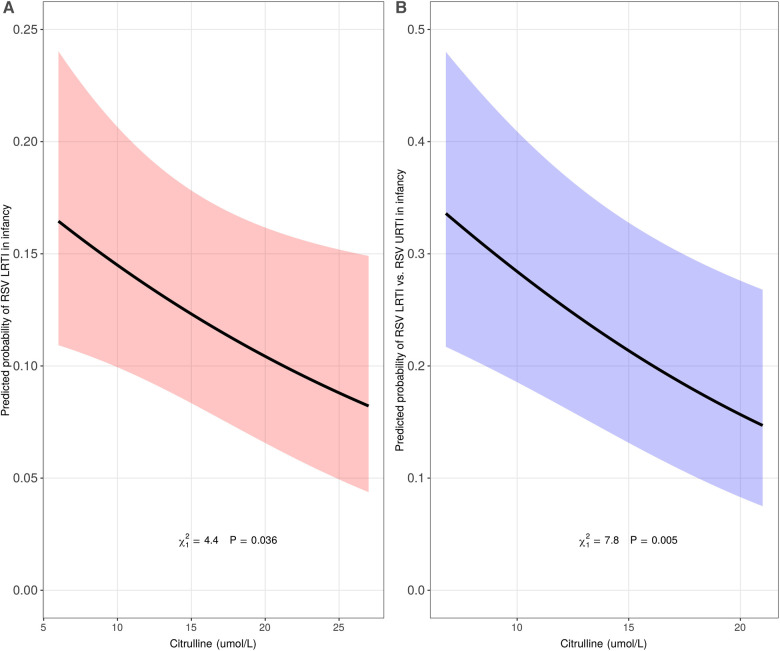
Association of citrulline concentration at birth with lower respiratory tract infection (LRTI) of all etiologies (panel A, *n* = 1746) and respiratory syncytial virus (RSV) LRTI (panel B, *n* = 912) in infancy in the discovery cohort. The predicted probability (y-axis) in panel A was calculated using a multivariable logistic regression model in the discovery cohort, INSPIRE, adjusting for infant sex, infant race/ethnicity, mode of delivery, maternal smoking during pregnancy, ever breastfed, maternal asthma, living siblings, daycare attendance during the first year of life, and all other identified metabolites (C2, C3, C5, C5:1, C6-DC, C10:1, C10:2, C16, C18:2, ASA, GLY, ORN, SUAC, and VAL). The predicted probability (y-axis) in panel B was calculated using a multivariable logistic regression model in a subset of infants in INSPIRE with either RSV upper respiratory tract infection (URTI, less severe) or RSV LRTI (more severe), adjusting for infant sex, infant race/ethnicity, mode of delivery, maternal smoking during pregnancy, ever breastfed, maternal asthma, living siblings, and daycare attendance during the first year of life.

During the first year of life, 25%, 5%, and 3% of INSPIRE, MAAP, and WISC infants reported having an LRTI, respectively. The relationships between the identified set of 15 leading metabolites and covariates and the log odds of LRTI in infancy and the pairwise correlations between the leading metabolites are depicted in Supplementary Figure S3, Supplementary Table S4, and Supplementary Figure S4. While several metabolites were moderately associated with LRTI in infancy in adjusted analyses (C2, C3, C6-DC, and SUAC; *p* < 0.1), citrulline was the only statistically significant metabolite (Supplementary Figure S5). A 5 umol/l (IQR) increase of citrulline concentration at birth was associated with decreased odds of LRTI in infancy in the discovery cohort (adjusted odds ratio [aOR] 0.83 [95% confidence interval (CI) 0.70–0.99], *p* = 0.04) (Figure 2, panel A). When assessed in the replication cohorts, the meta-analyzed effect also showed an inverse relationship but was not statistically significant [aOR 0.58 (95% CI 0.28–1.22), *p* = 0.15] (Supplementary Table S5).

In the analysis restricted to INSPIRE infants who were infected with RSV during infancy [*n* = 912 (176 with RSV LRTI, 736 with RSV URTI)], we consistently observed an inverse relationship between citrulline concentration at birth and odds of LRTI in infancy [aOR 0.74 (95% CI 0.60–0.91), *p* = 0.005] (Figure 2, panel B).

## Discussion

We observed a protective association of citrulline concentration at birth on risk of LRTI in infancy, where increased concentration (within the normal range) was associated with decreased risk. This association persisted when assessed among a subset of infants who were infected with RSV during the first year of life, identifying novel metabolic pathways in early life that may be involved in susceptibility to severe respiratory viral infection, including RSV infection.

Energy metabolism is highly regulated to meet the demands of the cell. Alterations in metabolic pathways have been linked to cell death and dysfunction, reactive oxygen species production, altered immune response, and enhanced inflammation (19). Severe forms of metabolic dysregulation (i.e., inborn errors of metabolism) are identified at birth through NBS. These disorders are caused by genetic mutations leading to defective enzymes, cofactors, or transporters in metabolic pathways, often resulting in toxic accumulation of metabolite intermediates (20, 21). Respiratory manifestations, particularly airway infections, are common among children with inherited metabolic diseases (22), indicating that dysregulation of energy metabolism may predispose children to respiratory illness. Milder forms of metabolic dysregulation have also been implicated in susceptibility to pulmonary infections through impaired immune responses and increased airway inflammation (23). For this reason, we assessed the association between the variability in normal infant metabolism and viral infection severity to provide novel insights on LRTI pathogenesis, to aid in the identification of those at risk, and identify new pathways that may modify immune regulation that could be targeted for prevention and treatment.

LRTIs are a major public health issue (1), and with the exception of antiviral agents for influenza and SARS-CoV-2, the only available infant treatment is supportive care (5). RSV is the most common respiratory viral etiology of severe LRTIs (4). RSV prevention products (i.e., RSV immunoprophylaxis) are currently primarily available in high income countries for a small subset of high-risk infants (24). However, most infant RSV-related deaths occur in low and middle income countries (25), and over 70% of RSV-related hospitalizations occur in term infants without known comorbidities (26). There are no maternal or infant RSV vaccines currently approved, although many are in clinical trials (27, 28). Citrulline is a readily available dietary supplement and supplementation has been shown to be both safe and well tolerated in infants (29). Although citrulline is primarily used for treating urea cycle defects, there is accumulating evidence to suggest that citrulline supplementation may have a wider therapeutic role (30), particularly for lung diseases (31). Our findings may have important clinical application and provide sufficient evidence to further understand the protective effect of citrulline on LRTI in experimental models. While we focused on citrulline (per our *a priori* statistical plan), the metabolites C2, C3, C6-DC, and SUAC showed modest associations with LRTI, and therefore, may also be of interest in future studies.

The role of citrulline in the regulation of immune function is increasingly being recognized. Citrulline is a nutritionally non-essential amino acid which plays an important role in arginine biosynthesis, the nitric oxide cycle, and the urea cycle (32). The metabolism of citrulline is unique in that it is not used by the intestine or taken up by the liver and, thus, bypasses splanchnic extraction (33). Impaired conversion of citrulline to arginine in argininosuccinate synthetase deficiency has been shown to result in impaired immunity, increased infection susceptibility, and decreased nitric oxide production in experimental models (34–36). Children who are critically ill have low plasma citrulline concentration, which is strongly correlated with severity of inflammation (37). L-citrulline supplementation has been shown to enhance T-cell function by promoting IL-10 and TGF-*β*1 production in infant rats (36). Citrulline and *Lactobacillus* probiotic supplementation have been found to have a synergistic, protective effect against pathogen adhesion (i.e., the first step of pathogen infection) in the intestinal tract, and, thus, may be beneficial for improving immunity (38). Citrulline-generating and utilizing enzymes have also been reported to increase in lung myeloid populations during infection, enhancing host defense (39). It is plausible that citrulline's role in the development of the host immune response is one pathway through which increased citrulline concentration at birth could protect against risk of LRTI in infancy. While citrulline is a precursor for arginine, we did not observe a significant association between arginine and LRTI in infancy. This may be due to the distinct role of citrulline in immune modulation and nitric oxide synthesis (36, 40).

Our study has many strengths, including our use of routinely collected and uniformly measured newborn metabolic data and birth cohorts in the ECHO-CREW consortium, including a population-based birth cohort of term, healthy infants for discovery and two additional study cohorts for replication. Although our replication analyses were not statistically significant, they were very small in comparison with the discovery cohort. Despite this, we demonstrated consistent effect sizes and directionality in independent cohorts from diverse populations, which increases the validity of our findings. As our replication cohorts were small, the non-significant finding may be due to low power.

There are some limitations to our study. Replication cohorts were challenging to find, and LRTI was not uniformly ascertained among the three study populations, which likely accounted for the large difference in LRTI incidence. As the INSPIRE cohort was designed to include surveillance for acute respiratory illnesses and respiratory tract infections during infancy, the incidence of LRTI in this population was similar to previously reported population-based estimates (41). LRTI was likely rare among infants in our two replication cohorts because there was no protocol to track these illnesses. However, this would result in misclassification among those considered as having no LRTI and would drive findings towards the null, which suggests our replication results are conservative. Additionally, while the discovery cohort was a population-based birth cohort representative of the region from which participants were recruited, overall, the cohorts included in this study were primarily of non-Hispanic ethnicity, which may limit the generalizability of our findings. As the etiologic viral agent of acute respiratory illnesses was not captured in our replication cohorts, we were unable to replicate the association between citrulline concentration at birth and LRTI during the first year of life among infants with RSV infection in these cohorts.

## Conclusions

We identified a protective association of newborn citrulline concentration on risk of LRTI during the first year of life. Our findings allow us to speculate on this amino acid's mechanistic role and importance during pregnancy or early infancy on later LRTI risk and identify an available novel target for LRTI prevention and possibly treatment, where none currently exist for most infants. These data provide support for further investigation of the mechanisms underlying this relationship with a goal toward clinical translation.

## Data Availability

The data analyzed in this study is subject to the following licenses/restrictions: The dataset includes confidential newborn screening data that cannot be shared without individual approvals from the Michigan Department of Health and Human Services, Tennessee Department of Health, and Wisconsin Laboratory of Hygiene Institutional Review Boards. These data were linked to potentially identifying and sensitive patient information of participants in 3 CREW birth cohorts. Access to confidential CREW data requires written authorization from the CREW study sponsor, The National Institutes of Health Environmental Influences on Child Health Outcomes Program, and a data request submitted to the CREW PI. Requests to access these datasets should be directed to: Tina Hartert, tina.hartert@vumc.org.
