# Supplementary material for: Association of citrulline concentration at birth with lower respiratory tract infection in infancy: Findings from a multi-site birth cohort study
Source: Front Pediatr. 2022 Oct 17;10:979777. doi: 10.3389/fped.2022.979777 (PMC9618869; doi:10.3389/fped.2022.979777)
Supplement: Supplementary file 1 [file DataSheet1.docx]

**Supplementary Material**

[Methods 2](#_Toc114126795)

[Study design and populations 2](#_Toc114126796)

[Newborn screening metabolic data collection 2](#_Toc114126797)

[Lower respiratory tract infection and demographic and clinical characteristics ascertainment 3](#_Toc114126798)

[Statistical analysis 3](#_Toc114126799)

[Power calculation 4](#_Toc114126800)

[References 5](#_Toc114126801)

[Figure legends 6](#_Toc114126802)

[Supplementary Table 1 7](#_Toc114126803)

[Supplementary Table 2 9](#_Toc114126804)

[Supplementary Table 3 10](#_Toc114126805)

[Supplementary Table 4. 12](#_Toc114126806)

[Supplementary Table 5. 14](#_Toc114126807)

# Methods

## Study design and populations

This multi-site cohort study included three birth cohorts from the NIH Environmental influences on Child Health Outcomes (ECHO) Children’s Respiratory and Environmental Workgroup (CREW) consortium, which consists of birth cohorts established to assess the influence of prenatal and/or early life exposures on the development of asthma and allergic diseases.(1) We utilized the largest of the three cohorts (Infant Susceptibility to Pulmonary Infections and Asthma following RSV Exposure [INSPIRE], n=1949) for the discovery phase and the smaller cohorts (Microbes, Allergy, Asthma and Pets study [MAAP], n=141; Wisconsin Infant Study Cohort [WISC], n=270) to replicate findings. We included enrolled infants with at least one year of follow-up whom we linked with newborn screening (NBS) blood metabolic data. These cohorts have been described previously.(2, 3) Briefly, INSPIRE is a population-based, longitudinal birth cohort of term, healthy infants enrolled shortly after birth from pediatric practices located in middle Tennessee from 2012-2014. MAAP is a longitudinal birth cohort of term infants born to women at Henry Ford Hospital in Detroit from 2014-2016 who were and were not pet owners. WISC is a longitudinal birth cohort of term infants with varying exposure to farm environments prenatally and during the first two years of life, enrolled from healthcare providers in rural Wisconsin from 2013-2019.

## Newborn screening metabolic data collection

Our primary exposures were metabolite concentrations at birth measured by NBS programs. NBS is a state-mandated public health service. Screening is performed by a health care professional within a strict time window (24-48 hours after birth) and involves collection of a few drops of blood on a blood spot card obtained through a heel stick. Samples are tested at state laboratories and the quantified results are then stored on servers at state public health departments.(4) NBS metabolic data include targeted measurement of amino acids, free carnitine, and acylcarnitines. Each state’s public health department is responsible for deciding which conditions, and corresponding metabolites, are included on the NBS panel based on evidence of net benefit of screening, availability of effective treatments, and screening capability of the state. Most states screen for the panel of conditions recommended by the U.S. Health Resources & Services Administration, using the same methodology,(5) while some states additionally screen for newer conditions.(4, 6) Metabolites measured in each cohort are listed in Supplementary Table 1.

Existing NBS metabolic data were provided for newborns with available data and metabolite concentrations within the normal range (INSPIRE: 99%, MAAP: 95%, WISC: 92% [Figure 1]) by the NBS programs at the Tennessee Department of Health, Michigan Department of Health and Human Services, and Wisconsin State Laboratory of Hygiene. These data were then linked with demographic and clinical data from each of the cohorts. Metabolite concentrations were quantitatively measured by tandem mass spectrometry (MS/MS) using the calculated ratio of the signal from each metabolite to the signal from the known amount of internal standard.(5) Repeat blood spot testing results were provided for a small subset of infants based on state protocols (INSPIRE: n=37 [2% of enrolled participants], MAAP: n=0, WISC: n=0). For infants with two viable samples collected (INSPIRE: n=30), the average value of each metabolite was calculated and used in the analyses. For the remaining seven infants in whom the primary collection occurred outside of the protocol time frame (i.e., <24 hours after birth) or the sample was processed >10 days after collection, the value for the second specimen was used.(7)

## Lower respiratory tract infection and demographic and clinical characteristics ascertainment

Our primary outcome was lower respiratory tract infection (LRTI) at any time during the first year of life. LRTI was ascertained by parental report, physician diagnosis, or medical record documentation of bronchiolitis or pneumonia during the first year of life and defined at age one-year dichotomously as LRTI yes or no. Data collection for ascertainment of the primary outcome within each cohort is summarized in Supplementary Table 2. Information on demographic characteristics (infant sex, infant race/ethnicity, maternal smoking during pregnancy, secondhand smoke exposure during the first year of life, daycare attendance during the first year of life, ever breastfed, maternal education, maternal marital status, residence during the first six months of life, number of living siblings, year of birth, and age at infant enrollment) and clinical characteristics (gestational age, birthweight, mode of delivery, maternal asthma, paternal asthma, maternal allergic rhinitis) of the cohorts were ascertained from questionnaires administered during the first year of life.

## Statistical analysis

We compared demographic and clinical characteristics between the cohorts using Kruskal-Wallis or Pearson χ^2^ test, as appropriate. Our *a priori* statistical plan utilized a common, pre-specified two-stage procedure(8) to identify LRTI-related metabolites in the discovery cohort (Supplementary Figure 1). In the first stage, we used elastic net to identify leading metabolites associated with LRTI in the first year of life. Elastic net regularization is an efficient method for selecting groups of correlated metabolites while performing variable selection and continuous shrinkage.(9) We included all metabolites in one penalized logistic regression model, which helped reduce the multiple metabolite testing burden. Elastic net is particularly beneficial in this study as it tends to recover the true model when the ratio of sample size to model covariates (e.g., targeted metabolites) is small, regardless of covariate correlation.(10) The optimal shrinkage was obtained by using 10-fold cross validation to find the best value of tuning parameters (λ), which minimize mean-squared prediction errors. Although there was some skewness and zero values, metabolite concentrations were generally well distributed (normally) (Supplementary Figure 2). As having normal distributions is not an assumption of logistic regression, we utilized raw, untransformed metabolite concentrations in the analyses. Pairwise correlation coefficients between the leading metabolites associated with LRTI in the first year of life were calculated using the Spearman rank correlation method.

In the second stage, we evaluated the selected metabolites in a multivariable logistic regression model simultaneously, while adjusting for *a priori* selected demographic and clinical characteristics previously shown to be associated with metabolism or infant LRTI (infant sex, infant race/ethnicity, mode of delivery, maternal smoking during pregnancy, ever breastfed, maternal asthma, number of living siblings, and daycare attendance during the first year of life). Model re-estimation including only elastic-net chosen metabolites is appropriate as it tends to reduce the number of false positives.(8) We calculated each metabolite’s adjusted odds ratio for an interquartile range [IQR] difference increase in its concentration. The IQR represents a meaningful difference of moving from lower to higher quartiles and may be compared across studies with different populations.

For each metabolite that remained significantly associated with LRTI after the second stage (p<0.05), we tested its association with LRTI in the replication cohorts using meta-analyzed logistic regression with a fixed-effects inverse variance model. In the replication analysis, our regression power was limited by sparse LRTI events. To avoid overfitting, we decided *a priori* to limit covariate adjustment to infant sex.

To provide additional insight into pathways involved in severity of respiratory syncytial virus (RSV) infection, the most common causal pathogen of LRTI in infancy,(11) we performed a sub-analysis among a subset of infants in the discovery cohort with either RSV upper respiratory tract infection (URTI, less severe) or RSV LRTI (more severe) identified through biweekly surveillance during RSV season (November-March) and serology at one year. RSV LRTI was defined by an RSV positive reverse transcription polymerase chain reaction (RT-PCR) test and health care provider diagnosis of LRTI at the time of the respiratory illness during the infant’s first RSV season. RSV URTI was defined by an RSV positive RT-PCR test and no health care provider diagnosis of LRTI at the time of the respiratory illness during the infant’s first RSV season or an RSV positive serology test at one year of age and no health care provider diagnosis of LRTI during the first year of life. We assessed the association between each metabolite identified in the primary analysis and RSV LRTI using multivariable logistic regression, adjusting for the same covariates included in stage two of the primary analysis. Data analyses were performed using R software, versions 3.6.1 and 4.0.4 (R Foundation for Statistical Computing, Vienna, Austria).

## Power calculation

We performed a power analysis to detect a conservative difference in citrulline concentration at birth (similar to the one we report in this study) associated with LRTI in infancy in our replication cohorts. PS software(12) and data from Supplementary Table 4 difference in mean citrulline concentrations at birth between infants with and without LRTI in the first year of life within INSPIRE were used to perform this power analysis. We used a 5% prevalence of LRTI from one of our replication cohorts (MAAP), and we evaluated power with a standard deviation that ranged from 1.0-5.0 and a 5% type I error rate. Replication cohorts’ power were estimated to be between 6-25%.

# References

1. Gern JE, Jackson DJ, Lemanske RF, Jr., Seroogy CM, Tachinardi U, Craven M, et al. The Children's Respiratory and Environmental Workgroup (Crew) Birth Cohort Consortium: Design, Methods, and Study Population. *Respiratory research* (2019) 20(1):115-. doi: 10.1186/s12931-019-1088-9.

2. Larkin EK, Gebretsadik T, Moore ML, Anderson LJ, Dupont WD, Chappell JD, et al. Objectives, Design and Enrollment Results from the Infant Susceptibility to Pulmonary Infections and Asthma Following Rsv Exposure Study (Inspire). *BMC Pulm Med* (2015) 15:45. Epub 2015/05/30. doi: 10.1186/s12890-015-0040-0.

3. Seroogy CM, VanWormer JJ, Olson BF, Evans MD, Johnson T, Cole D, et al. Respiratory Health, Allergies, and the Farm Environment: Design, Methods and Enrollment in the Observational Wisconsin Infant Study Cohort (Wisc): A Research Proposal. *BMC Res Notes* (2019) 12(1):423. Epub 2019/07/18. doi: 10.1186/s13104-019-4448-0.

4. Baby's First Test. Available from https://www.babysfirsttest.org/. Accessed August 29, 2022.

5. CLSI. Newborn Screening by Tandem Mass Spectrometry. 2nd Ed. CLSI Guideline NBS04. Wayne, Pa: Clinical and Laboratory Standards Institute; 2017.

6. Health Resources & Services Administration. Recommended Uniform Screening Panel. Available from https://www.hrsa.gov/advisory-committees/heritable-disorders/rusp/index.html. Accessed August 31, 2022.

7. Donovan BM, Ryckman KK, Breheny PJ, Gebretsadik T, Turi KN, Larkin EK, et al. Association of Newborn Screening Metabolites with Risk of Wheezing in Childhood. *Pediatric research* (2018). Epub 2018/06/13. doi: 10.1038/s41390-018-0070-4.

8. van de Geer S, Bühlmann P, Zhou S. The Adaptive and the Thresholded Lasso for Potentially Misspecified Models (and a Lower Bound for the Lasso). *Electronic Journal of Statistics* (2011) 5(none):688-749, 62.

9. Zou H, Hastie T. Regularization and Variable Selection Via the Elastic Net. *Journal of the Royal Statistical Society Series B (Statistical Methodology)* (2005) 67(2):301-20.

10. Honorio J, Jaakkola T. A Unified Framework for Consistency of Regularized Loss Minimizers. *Journal of Maching Learning Reserach: Workshop and Conference Proceedings* (2014) 32:136-44.

11. Piedra PA, Stark AR. Bronchiolitis in Infants and Children: Clinical Features and Diagnosis. (Accessed September 12, 2022. In: Uptodate [Internet]. Waltham, Ma: Uptodate. Available from: https://www.uptodate.com/contents/bronchiolitis-in-infants-and-children-clinical-features-and-diagnosis/print.

12. Dupont WD, Plummer WD, Jr. Power and Sample Size Calculations. A Review and Computer Program. *Controlled clinical trials* (1990) 11(2):116-28. Epub 1990/04/01. doi: 10.1016/0197-2456(90)90005-m.

# **Figure legends**

**Supplementary Figure 1. Diagram of *a priori* statistical plan.**

**Supplementary Figure 2. Newborn screening metabolite distributions in the discovery cohort (n=1746).**

**Supplementary Figure 3. Leading set of newborn screening metabolites (umol/L) and log odds of lower respiratory tract infection in infancy in the discovery cohort (n=1746).** P-values for testing associations were calculated using multivariable logistic regression, adjusting for infant sex, infant race/ethnicity, mode of delivery, maternal smoking during pregnancy, ever breastfed, maternal asthma, living siblings, daycare attendance during the first year of life, and all other identified metabolites.

**Supplementary Figure 4. Pairwise correlation between the leading metabolites associated with lower respiratory tract infection in the first year of life in the discovery cohort (n=1746).** Pairwise correlations were calculated using Spearman rank correlation.

**Supplementary Figure 5.** **Leading metabolites associated with lower respiratory tract infection (LRTI) in the first year of life in the discovery cohort (n=1746).** *p<0.05. Adjusted odds ratios were estimated using multivariable logistic regression adjusting for infant sex, infant race/ethnicity, mode of delivery, maternal smoking during pregnancy, ever breastfed, maternal asthma, living siblings, daycare attendance during the first year of life, and all other identified metabolites. Each metabolite’s adjusted odds ratio was calculated for an interquartile range difference increase in its concentration.

Supplementary Table 1. **Newborn screening (NBS) metabolites measured for each cohort.**

| **Metabolite Name (units)** | **Metabolite Abbreviation** | **Measurement collected on NBS panel** | | |
| --- | --- | --- | --- | --- |
|  |  | **INSPIRE** | **MAAP** | **WISC** |
| Free carnitine (umol/L) | C0 | X | X | X |
| **Acylcarnitines (umol/L)** |  |  |  |  |
| Acetylcarnitine | C2 | X | X | X |
| Propionylcarnitine | C3 | X | X | X |
| Butyrylcarnitine+Isobutyrylcarnitine | C4 | X | X | X |
| Isovalerylcarnitine +  Methylbutyrylcarnitine | C5 | X | X | X |
| Tiglylcarnitine | C5:1 | X | X | X |
| Hexanoylcarnitine | C6 | X | X | X |
| Methylglutarylcarnitine | C6-DC | X | X |  |
| Octanoylcarnitine | C8 | X | X | X |
| Octenoylcarnitine | C8:1 |  | X |  |
| Decanoylcarnitine | C10 | X | X | X |
| Decenoylcarnitine | C10:1 | X | X | X |
| Decadienoylcarnitine | C10:2 | X | X | X |
| Dodecanoylcarnitine | C12 |  | X |  |
| Dodecenoylcarnitine | C12:1 |  | X | X |
| Tetradecanoylcarnitine | C14 | X | X | X |
| 3-Hydroxytetradecanoylcarnitine | C14-OH | X | X | X |
| Tetradecenoylcarnitine | C14:1 | X | X | X |
| Tetradecadienoylcarnitine | C14:2 |  | X | X |
| Palmitoylcarnitine | C16 | X | X | X |
| 3-Hydroxypalmitoylcarnitine | C16-OH | X | X | X |
| Hexadecanedioylcarnitine | C16-DC |  |  | X |
| Palmitoleylcarnitine | C16:1 | X | X | X |
| 3-Hydroxypalmitoleylcarnitine | C16:1-OH |  |  | X |
| Stearoylcarnitine | C18 | X | X | X |
| 3-Hydroxystearoylcarnitine | C18-OH |  | X | X |
| Oleoylcarnitine | C18:1 | X | X | X |
| 3-Hydroxyoleoylcarnitine | C18:1-OH | X | X | X |
| Octadecenedioylcarnitine | C18:1-DC |  |  | X |
| Linoleoylcarnitine | C18:2 | X | X | X |
| 3-Hydroxylinoleoylcarnitine | C18:2-OH |  |  | X |
| **Amino Acids (umol/L)** |  |  |  |  |
| Alanine | ALA |  | X |  |
| Arginine | ARG | X | X |  |
| Argininosuccinate | ASA | X | X |  |
| Citrulline | CIT | X | X | X |
| Glycine | GLY | X | X |  |
| Leucine | LEU | X | X | X |
| Methionine | MET | X | X | X |
| Ornithine | ORN | X | X |  |
| Phenylalanine | PHE | X | X | X |
| Proline | PRO |  | X |  |
| Succinylacetone | SUAC | X | X |  |
| Tyrosine | TYR | X | X | X |
| Valine | VAL | X | X | X |
| **Total** |  | **33** | **40** | **34** |

Supplementary Table 2**. Data collection for ascertainment of the primary outcome of lower respiratory tract infection (LRTI) in the first year of life within each cohort.**

| **Cohort** | **Study Visit** | **Ascertainment of LRTI** |
| --- | --- | --- |
| **INSPIRE** | Enrollment visit^a^ | Parent reported |
|  | Respiratory illness visit^b^ | Physician diagnosis |
|  | 1-year visit | Parent reported |
| **MAAP** | Post-delivery visit | Parent reported |
|  | 6-month visit | Parent reported |
|  | 18-month visit | Parent reported |
|  | Health care encounter | Medical chart documentation of LRTI diagnosis during first year of life |
| **WISC** | 2-month visit | Parent reported |
|  | 6-month visit | Parent reported |
|  | 9-month visit | Parent reported |
|  | 12-month visit | Parent reported |
|  | Health care encounter | Medical chart documentation of LRTI diagnosis during first year of life |

^a^The mean (SD) of age at enrollment in was 2 months (2 months).

^b^Surveillance was performed every two weeks during the infant’s first respiratory syncytial virus season (November-March). In-person visits were performed based on parent report of symptoms or diagnosis of respiratory illness. Diagnosis was confirmed through physical exam by a healthcare provider.

Supplementary Table 3**.** **Newborn screening metabolite concentrations for infants with linked newborn screening data and non-missing outcome data.**

| **Metabolite**  **(umol/L)** | **INSPIRE**  **Mean (SD)** | **MAAP**  **Mean (SD)** | **WISC**  **Mean (SD)** |
| --- | --- | --- | --- |
| **Total sample size** | **1746** | **134** | **222** |
| Free carnitine (C0) | 19.41 (7.66) | 24.06 (8.98) | 17.03 (6.25) |
| Missing^a^ | 7 (0) | 0 (0) | 0 (0) |
| **Acylcarnitines** |  |  |  |
| C2 | 22.64 (8.41) | 24.33 (8.34) | 29.42 (9.55) |
| Missing^a^ | 7 (0) | 0 (0) | 0 (0) |
| C3 | 1.53 (0.64) | 1.92 (0.74) | 2.18 (0.82) |
| Missing^a^ | 7 (0) | 0 (0) | 0 (0) |
| C4 | 0.23 (0.10) | 0.27 (0.12) | 0.27 (0.13) |
| Missing^a^ | 7 (0) | 0 (0) | 0 (0) |
| C5 | 0.09 (0.04) | 0.11 (0.05) | 0.10 (0.05) |
| Missing^a^ | 12 (1) | 0 (0) | 0 (0) |
| C5:1 | 0.01 (0) | 0.01 (0) | 0.01 (0.01) |
| Missing^a^ | 7 (0) | 0 (0) | 0 (0) |
| C6 | 0.05 (0.02) | 0.06 (0.02) | 0.05 (0.03) |
| Missing^a^ | 7 (0) | 0 (0) | 0 (0) |
| C6-DC | 0.10 (0.03) | 0.12 (0.03) | -- |
| Missing^a^ | 8 (0) | 0 (0) | 222 (100) |
| C8 | 0.06 (0.03) | 0.07 (0.02) | 0.08 (0.03) |
| Missing^a^ | 7 (0) | 0 (0) | 0 (0) |
| C10 | 0.09 (0.04) | 0.10 (0.03) | 0.12 (0.05) |
| Missing^a^ | 7 (0) | 0 (0) | 0 (0) |
| C10:1 | 0.05 (0.01) | 0.05 (0.01) | 0.05 (0.02) |
| Missing^a^ | 7 (0) | 0 (0) | 0 (0) |
| C10:2 | 0.01 (0) | 0.01 (0) | 0.01 (0.01) |
| Missing^a^ | 7 (0) | 0 (0) | 0 (0) |
| C14 | 0.23 (0.08) | 0.26 (0.07) | 0.31 (0.10) |
| Missing^a^ | 7 (0) | 0 (0) | 0 (0) |
| C14:1 | 0.14 (0.07) | 0.15 (0.05) | 0.22 (0.10) |
| Missing^a^ | 7 (0) | 0 (0) | 0 (0) |
| C14-OH | 0.02 (0.01) | 0.02 (0.01) | 0.02 (0.01) |
| Missing^a^ | 7 (0) | 0 (0) | 0 (0) |
| C16 | 2.86 (0.97) | 3.22 (0.97) | 3.59 (1.01) |
| Missing^a^ | 7 (0) | 0 (0) | 0 (0) |
| C16:1 | 0.22 (0.09) | 0.24 (0.07) | 0.37 (0.12) |
| Missing^a^ | 7 (0) | 0 (0) | 0 (0) |
| C16-OH | 0.02 (0.01) | 0.03 (0.01) | 0.04 (0.03) |
| Missing^a^ | 7 (0) | 0 (0) | 0 (0) |
| C18 | 0.81 (0.28) | 0.95 (0.30) | 1.03 (0.33) |
| Missing^a^ | 7 (0) | 0 (0) | 0 (0) |
| C18:1 | 1.21 (0.40) | 1.33 (0.39) | 1.76 (0.53) |
| Missing^a^ | 7 (0) | 0 (0) | 0 (0) |
| C18:1-OH | 0.02 (0.01) | 0.02 (0.01) | 0.02 (0.02) |
| Missing^a^ | 7 (0) | 0 (0) | 0 (0) |
| C18:2 | 0.18 (0.11) | 0.21 (0.11) | 0.27 (0.14) |
| Missing^a^ | 7 (0) | 0 (0) | 0 (0) |
| **Amino Acids** |  |  |  |
| ARG | 9.12 (5.09) | 10.69 (5.83) | -- |
| Missing^a^ | 5 (0) | 0 (0) | 222 (100) |
| ASA | 0.07 (0.03) | 0.14 (0.07) | 0.25 (0.31) |
| Missing^a^ | 5 (0) | 0 (0) | 72 (32)^b^ |
| CIT | 12.56 (3.72) | 16.86 (4.08) | 12 .65 (4.08) |
| Missing^a^ | 5 (0) | 0 (0) | 0 (0) |
| GLY | 391.29 (92.42) | 489.73 (123.36) | -- |
| Missing^a^ | 5 (0) | 0 (0) | 222 (100) |
| LEU | 92.70 (23.55) | 106.03 (26.50) | 88.45 (22.26) |
| Missing^a^ | 5 (0) | 0 (0) | 0 (0) |
| MET | 19.55 (4.92) | 26.13 (6.70) | 19.32 (5.37) |
| Missing^a^ | 5 (0) | 0 (0) | 0 (0) |
| ORN | 70.00 (21.81) | 92.53 (29.87) | -- |
| Missing^a^ | 5 (0) | 0 (0) | 222 (100) |
| PHE | 49.87 (9.79) | 66.73 (12.93) | 52.14 (8.86) |
| Missing^a^ | 7 (0) | 0 (0) | 0 (0) |
| SUAC | 0.41 (0.11) | 0.54 (0.13) | -- |
| Missing^a^ | 7 (0) | 0 (0) | 222 (100) |
| TYR | 83.20 (33.00) | 112.17 (33.79) | 86.51 (35.89) |
| Missing^a^ | 7 (0) | 0 (0) | 0 (0) |
| VAL | 89.21 (23.80) | 10.69 (5.83) | 72.25 (17.32) |
| Missing^a^ | 7 (0) | 0 (0) | 0 (0) |

^a^Data are expressed as number of children with missing metabolite data (% of study population).

^b^Argininosuccinate (ASA) was added to Wisconsin newborn screening panel in late 2015; therefore, participants enrolled in WISC prior to 2016 were missing data for this metabolite.

-- Metabolite not collected.

Supplementary Table 4. **Association between leading metabolites and covariates and the log odds of lower respiratory tract infection (LRTI) in infancy within INSPIRE (n=1746).**

|  | **No LRTI**  **N (%)** | **LRTI**  **N (%)** | **OR**  **(95% CI)** | **aOR**  **(95% CI)** | **Adjusted p-value** |
| --- | --- | --- | --- | --- | --- |
| **Sample Size** | 1314 | 432 |  |  |  |
| C2^a^ | 22.86 (8.48) | 21.97 (8.17) | 0.88  (0.77-1.00) | 0.80  (0.64-1.02) | 0.07 |
| C3^a^ | 1.52 (0.63) | 1.55 (0.66) | 1.05  (0.92-1.20) | 1.18  (0.98-1.41) | 0.09 |
| C5^a^ | 0.09 (0.04) | 0.09 (0.04) | 1.08  (0.97-1.21) | 0.96  (0.82-1.12) | 0.58 |
| C5:1^a^ | 0.01 (0) | 0.01 (0) | 2.58  (0.89-7.50) | 2.42  (0.75-7.77) | 0.14 |
| C6-DC^a^ | 0.10 (0.03) | 0.10 (0.03) | 1.08  (0.95-1.23) | 1.17  (0.99-1.38) | 0.06 |
| C10:1^a^ | 0.05 (0.01) | 0.05 (0.02) | 1.11  (0.96-1.28) | 1.08  (0.92-1.28) | 0.35 |
| C10:2^a^ | 0.01 (0) | 0.01 (0) | 2.67  (0.86-8.29) | 1.59  (0.43-5.86) | 0.48 |
| C16^a^ | 2.92 (0.98) | 2.81 (0.94) | 0.86  (0.75-0.99) | 0.85  (0.69-1.05) | 0.14 |
| C18:2^a^ | 0.18 (0.10) | 0.19 (0.11) | 1.05  (0.95-1.16) | 1.03  (0.90-1.18) | 0.64 |
| ASA^a^ | 0.07 (0.03) | 0.07 (0.03) | 1.07  (0.96-1.21) | 1.02  (0.90-1.16) | 0.77 |
| CIT^a^ | 12.66 (3.76) | 12.26 (3.59) | 0.86  (0.74-1.00) | 0.83  (0.70-0.99) | 0.04 |
| GLY^a^ | 390.63 (92.42) | 939.28 (92.48) | 1.04  (0.91-1.19) | 1.12  (0.94-1.34) | 0.19 |
| ORN^a^ | 69.51 (21.21) | 71.46 (23.51) | 1.11  (0.98-1.26) | 1.07  (0.89-1.29) | 0.50 |
| SUAC^a^ | 0.40 (0.11) | 0.42 (0.12) | 1.15  (1.02-1.29) | 1.13  (0.99-1.29) | 0.07 |
| VAL^a^ | 88.75 (23.57) | 90.61 (24.48) | 1.09  (0.96-1.24) | 1.08  (0.91-1.28) | 0.37 |
| Infant sex |  |  |  |  | 0.07 |
| Male | 679 (52) | 241 (56) | Reference |  |  |
| Female | 635 (48) | 191 (44) | 0.85  (0.68-1.05) | 0.80  (0.63-1.01) |  |
| Infant race/ethnicity |  |  |  |  | 0.12 |
| Non-Hispanic White | 849 (65) | 271 (63) | Reference |  |  |
| Non-Hispanic Black | 238 (18) | 75 (17) | 1.00  (0.95-1.05) | 1.07  (0.75-1.54) |  |
| Hispanic | 106 (8) | 42 (10) | 1.04  (0.97-1.12) | 1.60  (1.06-2.41) |  |
| Other | 121 (9) | 44 (10) | 1.03  (0.96-1.10) | 1.28  (0.86-1.90) |  |
| Mode of delivery |  |  |  |  | 0.55 |
| Vaginal | 906 (69) | 296 (69) | Reference |  |  |
| Cesarean | 408 (31) | 136 (31) | 1.02  (0.81-1.29) | 0.92  (0.72-1.19) |  |
| Maternal smoking during pregnancy | 190 (14) | 104 (24) | 1.88  (1.43-2.45) | 1.94  (1.44-2.61) | <0.001 |
| Ever breastfed | 1071 (82) | 331 (77) | 0.83  (0.64-1.09) | 1.03  (0.74-1.43) | 0.86 |
| Maternal asthma | 244 (19) | 100 (23) | 1.32  (1.01-1.72) | 1.33  (1.00-1.76) | 0.05 |
| Living siblings |  |  |  |  | 0.003 |
| 0 | 491 (37) | 112 (26) | Reference |  |  |
| 1 | 398 (30) | 147 (34) | 1.09  (1.03-1.14) | 1.54  (1.15-2.06) |  |
| ≥2 | 425 (32) | 173 (40) | 1.11  (1.06-1.16) | 1.60  (1.19-2.15) |  |
| Daycare attendance during the first year of life | 410 (31) | 170 (39) | 1.54  (1.23-1.94) | 1.81  (1.41-2.30) | <0.001 |

SD, standard deviation; OR, odds ratio; CI, confidence interval.

^a^Data are expressed as mean (standard deviation).

Adjusted model: overall chi-square statistic 89.3, likelihood ratio fit statistic (null is intercept term only), p-value <0.001

Odds ratios and adjusted odds ratios were estimated for interquartile range increases in metabolite concentrations at birth (C2=10.00, C3=0.76, C5=0.04, C5:1=0.03, C6-DC=0.04, C10:1=0.02, C10:2=0.03, C16=1.19, C18:2=0.10, ASA=0.03, CIT=5.00, GLY=115.00, ORN=26.00, SUAC=0.12, VAL=28.00). Odds ratios were estimated using univariate logistic regression models. Adjusted odds ratios and corresponding p-values were estimated using multivariable logistic regression adjusting for all covariates and metabolites listed in the table.

| **Cohort** | **Number of infants with LRTI/total sample size** | **OR**  **(95% CI)** | **aOR**  **(95% CI)** | **Adjusted**  **p-value** |
| --- | --- | --- | --- | --- |
| **Discovery** |  |  |  |  |
| INSPIRE | 432/1746 | 0.86 (0.74-1.00) | 0.83 (0.70-0.99) | 0.04* |
| **Replication** |  |  |  |  |
| MAAP | 7/134 | 0.79 (0.29-2.13) | 0.70 (0.26-1.88) | 0.48 |
| WISC | 7/222 | 0.43 (0.14-1.34) | 0.46 (0.15-1.38) | 0.09 |
| MAAP and  WISC  meta-analyzed | 14/356 | 0.61 (0.29-1.28) | 0.58 (0.28-1.22) | 0.15 |

Supplementary Table 5. **Association between citrulline concentration at birth and lower respiratory tract infection (LRTI) in the first year of life within the study cohorts.**

OR, odds ratio; CI, confidence interval; aOR, adjusted odds ratio.

*Significant (α < 0.05)

Odds ratios and adjusted odds ratios were estimated for a 5 umol/L (interquartile range) increase of citrulline concentration at birth. Odds ratios were estimated using univariate logistic regression models. The adjusted odds ratio and corresponding p-value for INSPIRE was estimated using multivariable logistic regression adjusting for infant sex, infant race/ethnicity, mode of delivery, maternal smoking during pregnancy, ever breastfed, maternal asthma, living siblings, daycare attendance during the first year of life, and all other identified metabolites. Adjusted odds ratios and corresponding p-values for MAAP and WISC were estimated using multivariable logistic regression adjusting for infant sex. Meta-analysis was performed using a fixed-effects inverse variance model.
