# Supplementary figures and images for: Association of citrulline concentration at birth with lower respiratory tract infection in infancy: Findings from a multi-site birth cohort study
Source: Front Pediatr. 2022 Oct 17;10:979777. doi: 10.3389/fped.2022.979777 (PMC9618869; doi:10.3389/fped.2022.979777)

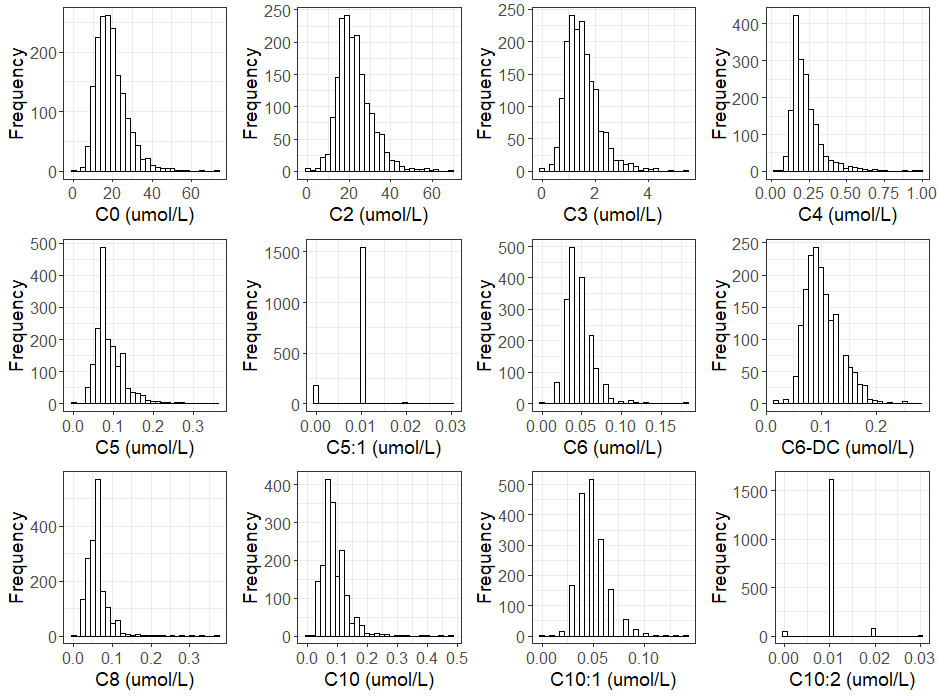

Supplement: Supplementary file 2 [file Image1.jpeg]

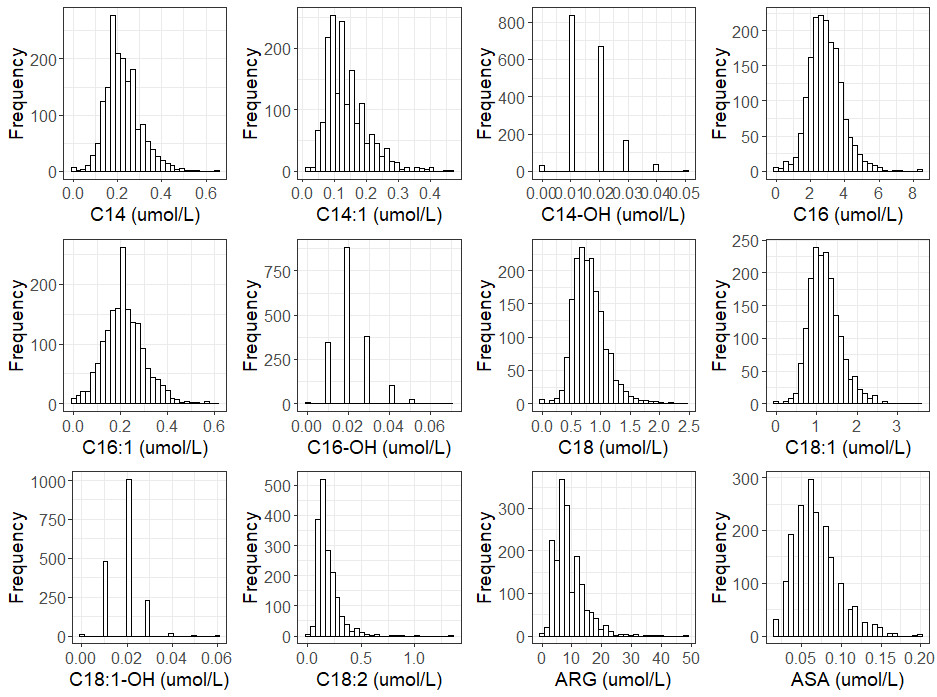

Supplement: Supplementary file 3 [file Image2.jpeg]

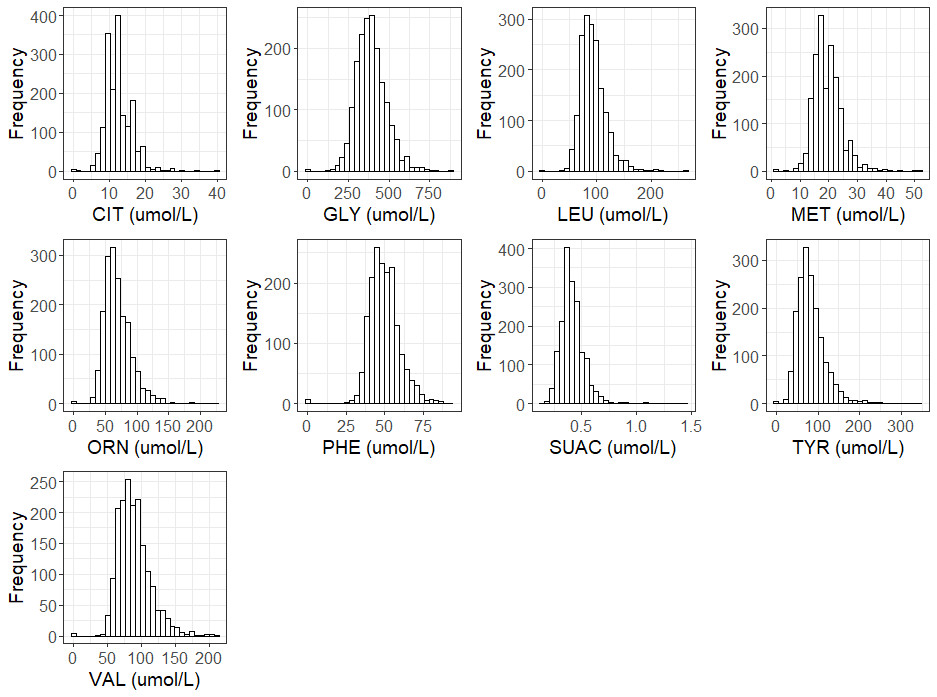

Supplement: Supplementary file 4 [file Image3.jpeg]

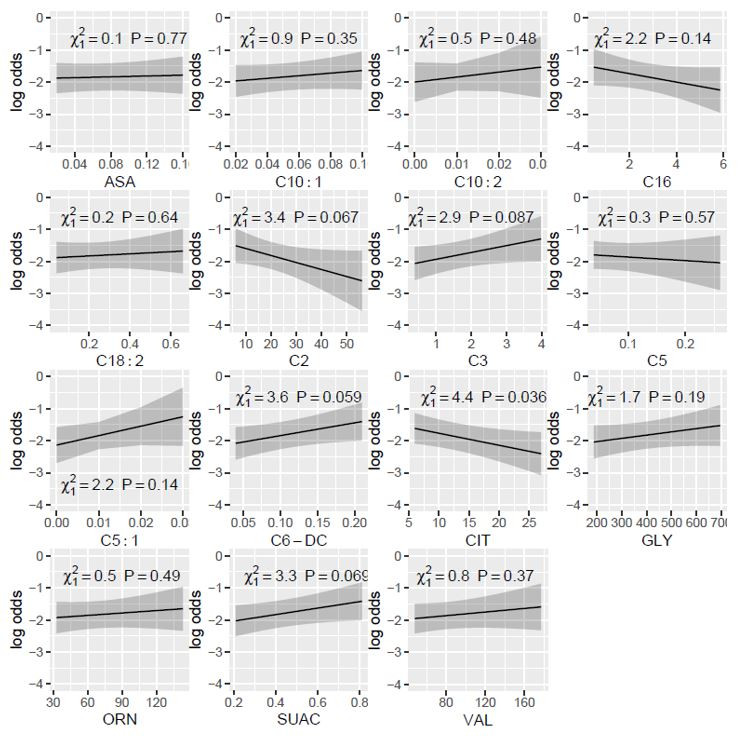

Supplement: Supplementary file 5 [file Image4.jpeg]

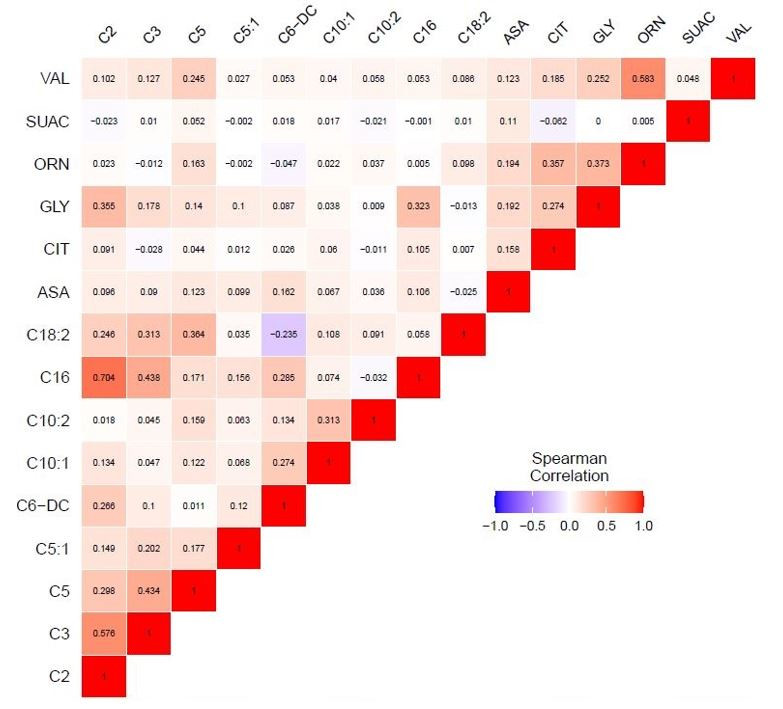

Supplement: Supplementary file 6 [file Image5.jpeg]

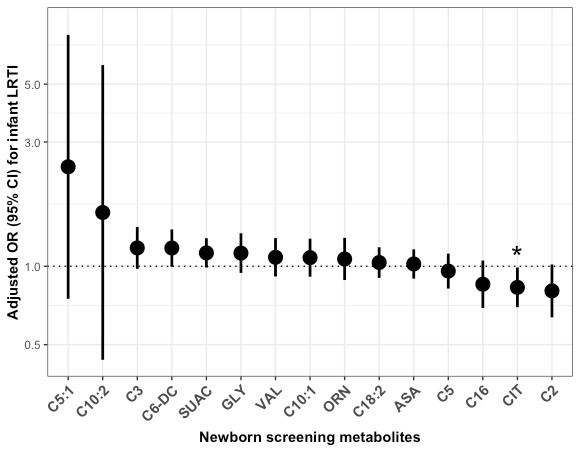

Supplement: Supplementary file 7 [file Image6.jpeg]

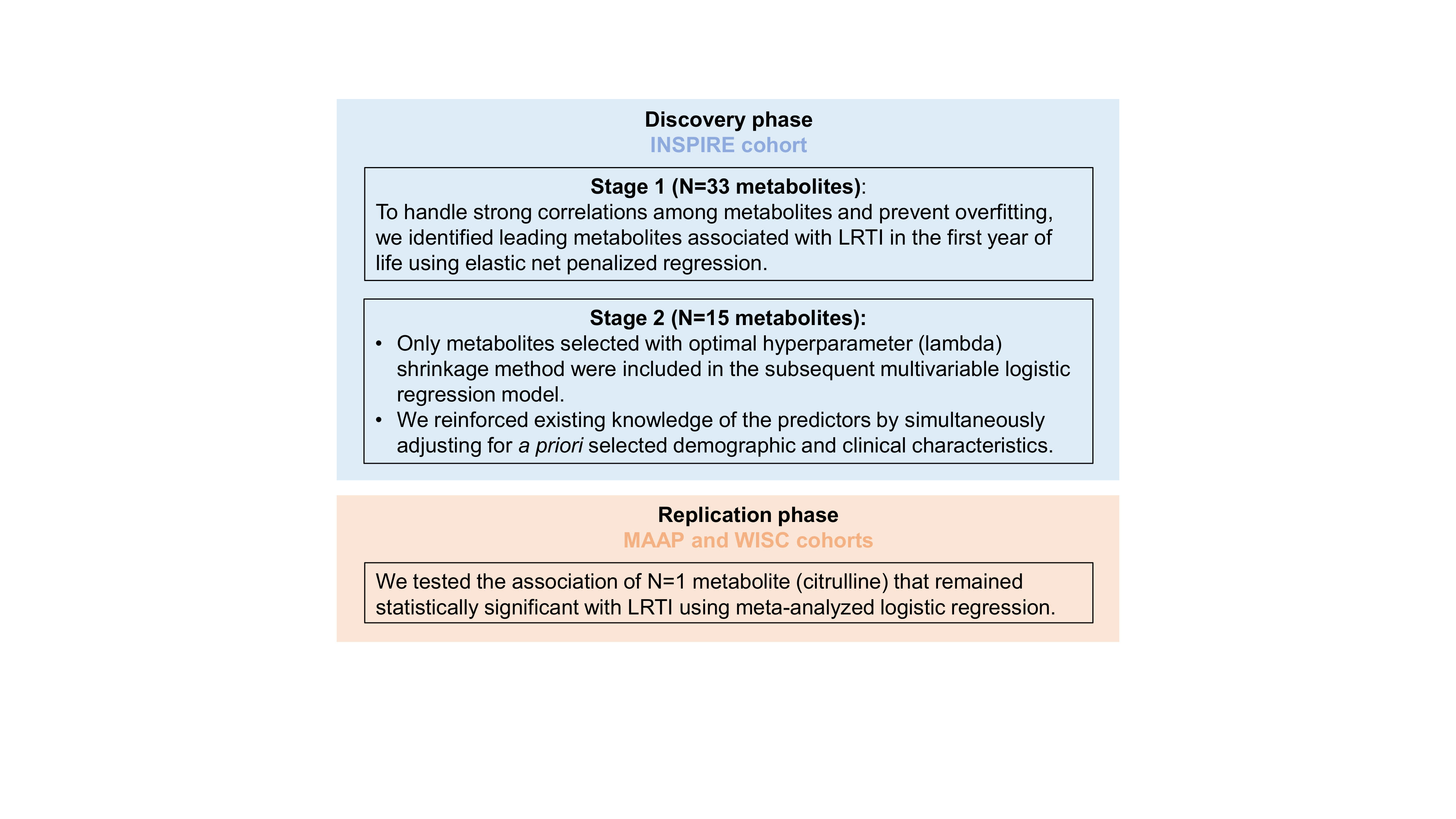

Supplement: Supplementary file 8 [file Image7.jpeg]
